# Reactive astrocyte-driven epileptogenesis is induced by microglia initially activated following status epilepticus

**DOI:** 10.1172/jci.insight.135391

**Published:** 2021-05-10

**Authors:** Fumikazu Sano, Eiji Shigetomi, Youichi Shinozaki, Haruka Tsuzukiyama, Kozo Saito, Katsuhiko Mikoshiba, Hiroshi Horiuchi, Dennis Lawrence Cheung, Junichi Nabekura, Kanji Sugita, Masao Aihara, Schuichi Koizumi

**Affiliations:** 1 Department of Neuropharmacology, Interdisciplinary Graduate School of Medicine,; 2 Department of Pediatrics, Faculty of Medicine, and; 3 Yamanashi GLIA Center, Interdisciplinary Graduate School of Medicine, University of Yamanashi, Yamanashi, Japan.; 4 Department of Neurology, Graduate School of Medical Science, Kyoto Prefectural University of Medicine, Kyoto, Japan.; 5 Shanghai Institute for Advanced Immunochemical Studies, ShanghaiTech University, Shanghai, China.; 6 Division of Homeostatic Development, National Institute for Physiological Sciences, Okazaki, Japan.

**Keywords:** Neuroscience, Calcium signaling, Epilepsy

## Abstract

Extensive activation of glial cells during a latent period has been well documented in various animal models of epilepsy. However, it remains unclear whether activated glial cells contribute to epileptogenesis, i.e., the chronically persistent process leading to epilepsy. Particularly, it is not clear whether interglial communication between different types of glial cells contributes to epileptogenesis, because past literature has mainly focused on one type of glial cell. Here, we show that temporally distinct activation profiles of microglia and astrocytes collaboratively contributed to epileptogenesis in a drug-induced status epilepticus model. We found that reactive microglia appeared first, followed by reactive astrocytes and increased susceptibility to seizures. Reactive astrocytes exhibited larger Ca^2+^ signals mediated by IP_3_R2, whereas deletion of this type of Ca^2+^ signaling reduced seizure susceptibility after status epilepticus. Immediate, but not late, pharmacological inhibition of microglial activation prevented subsequent reactive astrocytes, aberrant astrocyte Ca^2+^ signaling, and the enhanced seizure susceptibility. These findings indicate that the sequential activation of glial cells constituted a cause of epileptogenesis after status epilepticus. Thus, our findings suggest that the therapeutic target to prevent epilepsy after status epilepticus should be shifted from microglia (early phase) to astrocytes (late phase).

## Graphical abstract

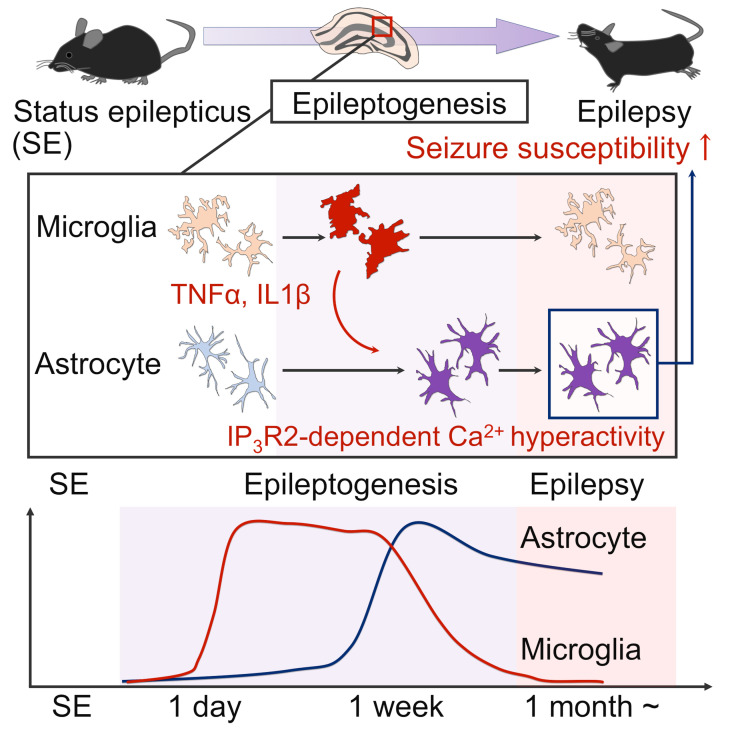

## Introduction

Epileptogenesis, i.e., the process leading to epilepsy, is a common sequel of brain insults such as brain injury, cerebrovascular disease, or status epilepticus (SE; refs. 1, 2). Such brain insults are typically followed by a latent period, during which the brain undergoes a cascade of morphological and functional changes over month to years prior to the onset of chronic epilepsy (3, 4). Extensive activation of glial cells, including microglia and astrocytes, has been well documented during this latent period in various animal models of epilepsy (5–7). Although the association of pathology with reactive glial cells is widely recognized, it is unclear whether such microglial and astrocytic activation constitutes primary causes of epilepsy or rather represents the results of repeated seizures. Moreover, the potential for these reactive glial cells to comprise candidates for epileptogenesis raises the further mechanistic question regarding whether activated glial cells might contribute to epileptogenesis independently or collaboratively.

In chemoconvulsant-induced epilepsy models, microglia are activated and produce proinflammatory mediators immediately after seizure onset (8). Activated microglia can decrease the seizure threshold in animal models by releasing proinflammatory molecules with neuromodulatory properties (9). Notably, the extent of microglial activation correlates with the seizure frequency in human drug-resistant epilepsy (10). Alternatively, such microglial activation may not persist chronically. For example, proinflammatory molecules are detectable in microglia after a seizure but the expression diminishes after several hours (11). Furthermore, although the activation of microglia is well characterized, it is unclear whether these activated microglia affect developing epileptogenic processes directly or through the modulation of other cells, such as subsequent astrocytic activation.

Reactive astrogliosis is also one of the most common pathological features in epilepsy and other brain insults (12, 13). Although reactive astrogliosis is considered the consequence of repetitive seizures, some evidence that reactive astrocytes may be responsible for repetitive seizures is available. In the epileptic brain, reactive astrocytes exhibit physiological and molecular changes, such as reduced inward rectifying K^+^ current (14), changes in transporters (15), release of gliotransmitters (16), or uncoupling of gap junction (17), that may underlie neuronal hyperexcitability (18). Although astrocytes do not exhibit prominent electrical excitability as observed in neurons, they are able to dynamically regulate calcium using internal stores (19, 20). Calcium transients in astrocytes are thought to modulate the release of a number of gliotransmitters that could influence synaptic function, synapse formation (21–24), and neural circuit excitability (25–27). In particular, several previous studies showed that astrocyte calcium activity could contribute to excitotoxic neuronal death through glutamate release after SE (28, 29). However, the functional changes, including Ca^2+^ signaling of reactive astrocytes after SE and their causal roles in epileptogenesis, remain largely uncertain.

To evaluate the role of interglial communication between different types of glial cells in the process of epileptogenesis, we assessed the spatiotemporal dynamics of glial activation after SE. Using cell type–specific manipulation, we show that relative alterations of both, microglia and astrocytes, play causal roles in epileptogenesis. Moreover, reactive glia are temporally distinct and collaboratively contribute to epileptogenesis. Reactive microglia appear first and induce reactive astrocytes in the hippocampus after SE. These reactive astrocytes present larger IP_3_R2-mediated Ca^2+^ signals, which are essential for induction of the increased seizure susceptibility after SE. We clearly demonstrate that the inhibition of microglial activation reduced astrogliosis, aberrant astrocytic Ca^2+^ signaling, and seizure susceptibility. We therefore conclude that the sequential activation of glial cells, i.e., the initial activation of microglia followed by astrocytic activation, was a cause of epileptogenesis after SE.

## Results

### Astrocytic activation followed microglial activation after SE.

**Figure 1:**
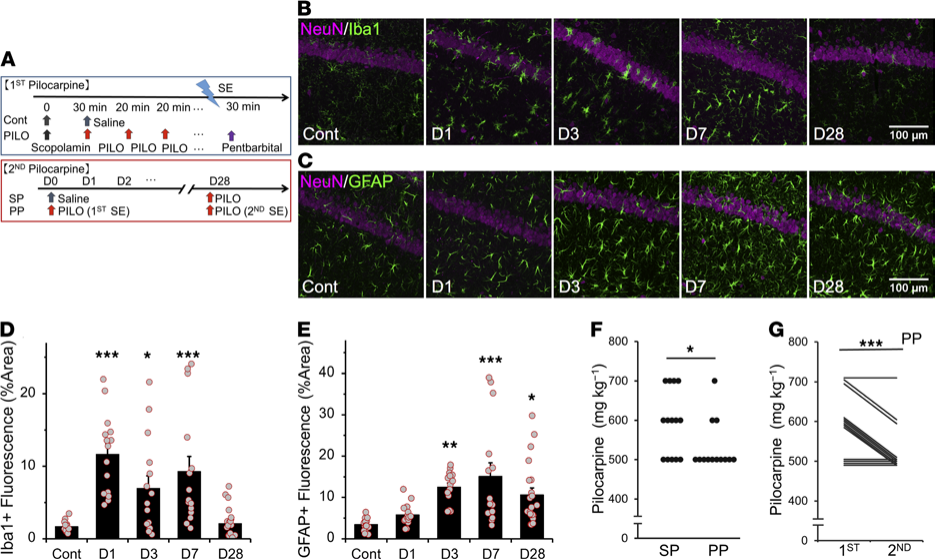
Astrogliosis is observed after microglial activation after SE. (**A**) As shown in the experimental protocols, mice were administered pilocarpine to achieve stage 5 seizures. The second SE was induced using the same protocol 4 weeks after the first SE. Mice were injected with saline (SP) or pilocarpine (PP) at 8 weeks of age followed by an injection of pilocarpine at 12 weeks of age. (**B** and **C**) Representative microphotographs showing the spatiotemporal characteristics of Iba1 (**B**) or GFAP (**C**) expression in CA1 after SE. Fifteen images were captured per z-stack image (0.5 μm step). (**D** and **E**) Quantification of the temporal profile of Iba1^+^ microglia (*n =* 5 mice) (**D**) or GFAP^+^ astrocytes (*n* = 5 [control and days 1, 3, and 7] and 7 [day 28] mice) (**E**) after SE (*P < 0.05, **P < 0.01 vs. control, 1-way ANOVA [***P < 0.001] with Dunnett’s test). (**F**) Dot plots showing dose of pilocarpine required for the induction of the second SE (*n =* 14 and 13 mice; *P < 0.05, Mann–Whitney *U* test). (**G**) Scatter plot showing dose of pilocarpine required for the induction of the first (at 8 weeks of age) and second (at 12 weeks of age) SE in the PP group (*n =* 13 mice; **P < 0.01, Wilcoxon signed-rank test). Values represent mean ± SEM. SE, status epilepticus; Cont, control.

To determine the contributions of glial cells to epileptogenesis, we used the pilocarpine model of epilepsy in mice, a model known to be highly isomorphic with human temporal lobe epilepsy (30, 31). Repeated low doses of pilocarpine (100 mg/kg) were injected i.p. until the onset of SE (Figure 1A). This ramping protocol has been shown to reduce mortality after SE (32, 33). To investigate how glial cell activation affects the epileptogenic process, we first examined the spatiotemporal pattern of microglial and astrocytic activation in the hippocampus after SE. We initially assessed microglial and astrocytic activation with IHC using cell type–specific activation markers on days 1, 3, 7, and 28 after SE (Figure 1, B and D). The area of Iba1^+^ microglia was significantly increased in CA1 on days 1–7 after SE, which was followed by an increase in the area of GFAP^+^ astrocytes in CA1 on days 3–28 after SE (Figure 1, C and E).

Of the 29 animals treated with pilocarpine, 10 survived the treatment and received a second treatment, 4 weeks after the first SE, to examine whether the first SE increased seizure susceptibility (the lethality in the first SE was 55.2%). A lower dose of pilocarpine was required for the induction of the second SE in mice with prior exposure to pilocarpine-induced SE at 8 weeks of age (PP) compared with mice without such exposure (mice with prior exposure to saline [SP mice]; Figure 1F). In addition, a lower dose of pilocarpine was required for the induction of the second SE compared with the first SE (Figure 1G). To measure the ictal and the interictal epileptiform activity, we performed EEG recordings of the left CA1 area of the dorsal hippocampus. Interictal spikes significantly increased on days 7 and 28 after SE (Supplemental Figure 1, A, B, C, and F; supplemental material available online with this article; https://doi.org/10.1172/jci.insight.135391DS1). These data indicated that the first SE increased seizure susceptibility even 4 weeks after the first SE. A comparison with the results in Figure 1 suggested that the temporal pattern of astrocyte activation, rather than that of microglia, correlated well with the increase of seizure susceptibility.

### Ca^2+^ hyperactivity via IP_3_R2 in reactive astrocytes after SE.

**Figure 2:**
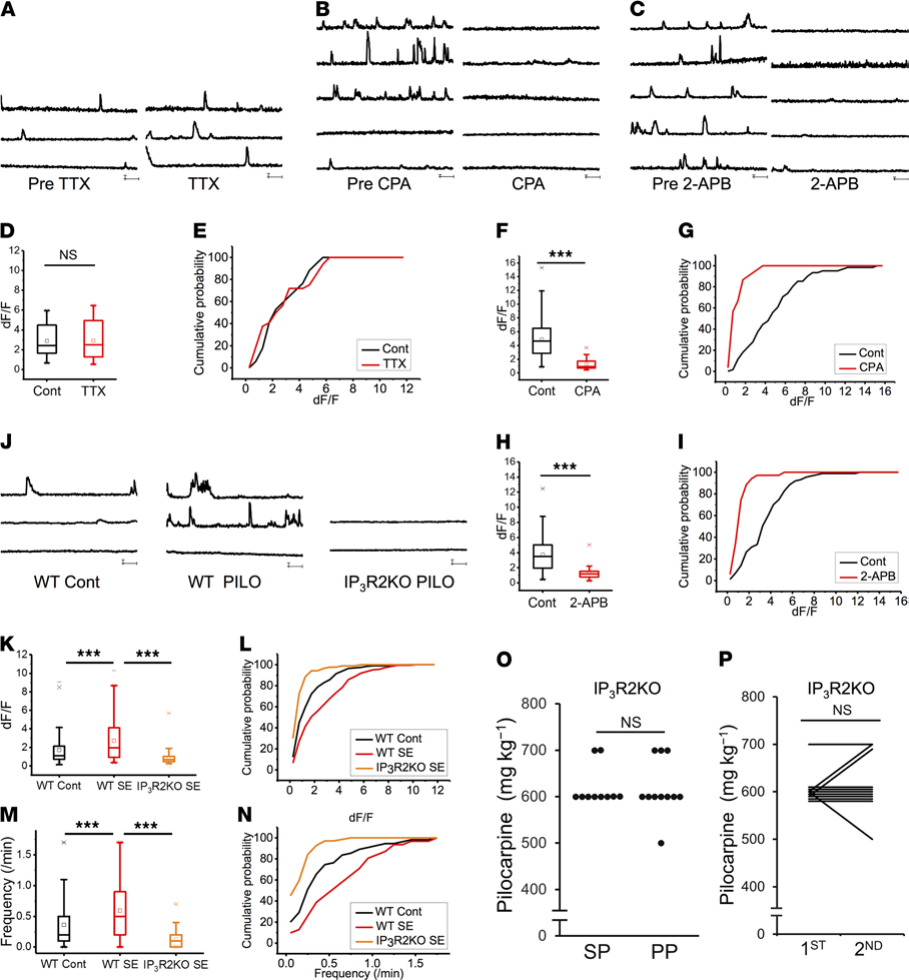
Reactive astrocytes exhibit IP_3_R2-mediated Ca^2+^ hyperactivity, which is essential for epileptogenesis. (**A**–**C**) Ca^2+^ dynamics of astrocytes approximately 4 weeks after SE in the CA1 stratum radiatum region in Glast-CreERT2 flx-GCaMP3 mice before and after TTX (1 μM) (**A**), CPA (20 μM) (**B**), and 2-APB (100 μM) (**C**) application. (**D**–**I**) Box plots showing amplitudes of Ca^2+^ signals before and after TTX (1 μM) (**D**), CPA (20 μM) (**F**), and 2-APB (100 μM) (**H**) application (*n* = 2 mice and 10 cells [**D**], 13 cells [**F**], and 14 cells [**H**]; ***P < 0.001, unpaired *t* test). Cumulative probability plots showing amplitudes (dF/F) of Ca^2+^ signals before and after TTX (NS *P* > 0.05, Kolmogorov–Smirnov test) (**E**), CPA (*P* < 0.001, Kolmogorov–Smirnov test) (**G**), and 2-APB (*P* < 0.001, Kolmogorov–Smirnov test) (**I**) application. (**J**) Astrocytic Ca^2+^ dynamics by Fluo4 in the CA1 stratum radiatum region in WT control, WT after SE, and IP_3_R2-KO mice after SE. (**K**–**N**) Box plots showing Ca^2+^ signal amplitudes (dF/F) (**K**) and frequency (**M**) (*n* = 57 cells and 2 mice [control], 32 cells and 2 mice [SE], and 85 cells and 3 mice [IP3R2KO SE]; ***P < 0.001, unpaired *t* test). Cumulative probability plots showing Ca^2+^ signal amplitudes (dF/F) (**L**) and frequency (**N**) (*P* < 0.001, Kolmogorov–Smirnov test). (**O**) Dot plots showing dose of pilocarpine required for the induction of the second SE in IP_3_R2-KO mice. Mice were injected with saline (SP) or pilocarpine (PP) at 8 weeks of age followed by an injection of pilocarpine at 12 weeks of age (*n =* 10 mice; NS *P* > 0.05, Mann–Whitney *U* test). (**P**) Scatter plot showing dose of pilocarpine required for the induction of the first (at 8 weeks of age) and second (at 12 weeks of age) SE in the PP group regarding IP3R2-KO mice (*n =* 10 mice; NS *P* > 0.05, Wilcoxon signed-rank test). Note: The first pilocarpine did not affect the dose required for the second SE in IP_3_R2-KO; see Figure 1G. TTX, tetrodotoxin; CPA, cyclopiazonic acid; 2-APB, 2-aminoethoxydiphenyl borate; Cont, control.

To examine the SE-induced functional changes in astrocytes, Ca^2+^ imaging was performed from hippocampal slices prepared from WT and Glast-CreERT2 flx-GCaMP3 mice (34, 35). Astrocytes displayed significantly larger Ca^2+^ signals approximately 4 weeks after SE in somata (Figure 2, J–L, and Supplemental Video 1). To test whether hyperactivity of astrocytes is influenced by neuronal hyperactivity, we blocked neuronal transmission by topically applying the voltage-gated sodium channel blocker tetrodotoxin (TTX; 1 μM). TTX did not affect the amplitude of astrocytic Ca^2+^ signals (Figure 2, A, D, and E, and Supplemental Video 2).

To elucidate the molecular mechanisms involved in astrocytic Ca^2+^ hyperactivity, we applied cyclopiazonic acid (CPA; 20 μM) to deplete intracellular calcium stores. CPA significantly reduced the amplitude of astrocytic Ca^2+^ signals after SE (Figure 2, B, F, and G, and Supplemental Video 3). Then, we applied the membrane-permeable IP_3_ receptor antagonist 2-aminoethoxydiphenyl borate (2-APB; 100 μM). 2-APB also significantly reduced the amplitude of astrocytic Ca^2+^ signals after SE (Figure 2, C, H, and I, and Supplemental Video 4). To confirm that astrocytic Ca^2+^ hyperactivity is completely dependent on the IP_3_ receptor, we performed Ca^2+^ imaging in IP_3_R2-KO mice (36). The amplitude of astrocytic Ca^2+^ signals after SE was significantly decreased in IP_3_R2-KO mice compared with that in WT mice (Figure 2, J–N). The frequency of astrocytic Ca^2+^ signals after SE was also significantly decreased in IP_3_R2-KO mice (Figure 2, M and N, and Supplemental Video 1). These results suggested that astrocytic Ca^2+^ hyperactivity after SE was dependent on IP_3_R2-mediated Ca^2+^ release from internal stores.

### IP_3_R2-KO mice exhibited rescue of the increased seizure susceptibility.

To clarify the role of astrocytic Ca^2+^ hyperactivity after SE in epileptogenesis, we investigated seizure susceptibility after SE in IP_3_R2-KO mice (36). No differences in the dose of pilocarpine required for the induction of the first SE were observed between IP_3_R2-KO and WT mice (Figure 1F and Figure 2O). These data indicate that IP_3_R2-mediated Ca^2+^ signaling in astrocytes did not alter the acute responses to pilocarpine.

In IP_3_R2-KO mice, the area of Iba1^+^ microglia was significantly increased in CA1 on day 1 after SE, suggesting that microglial activation after SE was comparable in IP_3_R2-KO and WT mice (Supplemental Figure 2). However, there was no significant change in the dose of pilocarpine required for the induction of the second SE in SP compared with PP mice (Figure 2O). Of the 16 animals treated with pilocarpine, 10 survived and received the second treatment (the lethality in the first SE was 37.5%.) There was no significant change in the dose of pilocarpine required for the induction of the first and second SE in IP_3_R2-KO mice (Figure 2P). In controlled conditions, there was no significant change in the number of interictal spikes in IP_3_R2-KO mice compared with WT mice (Supplemental Figure 1F). In addition, interictal spikes were significantly reduced in IP_3_R2-KO mice on day 28 after SE compared with those in WT mice (Supplemental Figure 1, E and F). These results suggest that IP_3_R2-mediated astrocytic Ca^2+^ hyperactivity was essential for the induction of the increased seizure susceptibility after SE.

### Microglia inhibition reduced activated astrocyte morphology.

**Figure 3:**
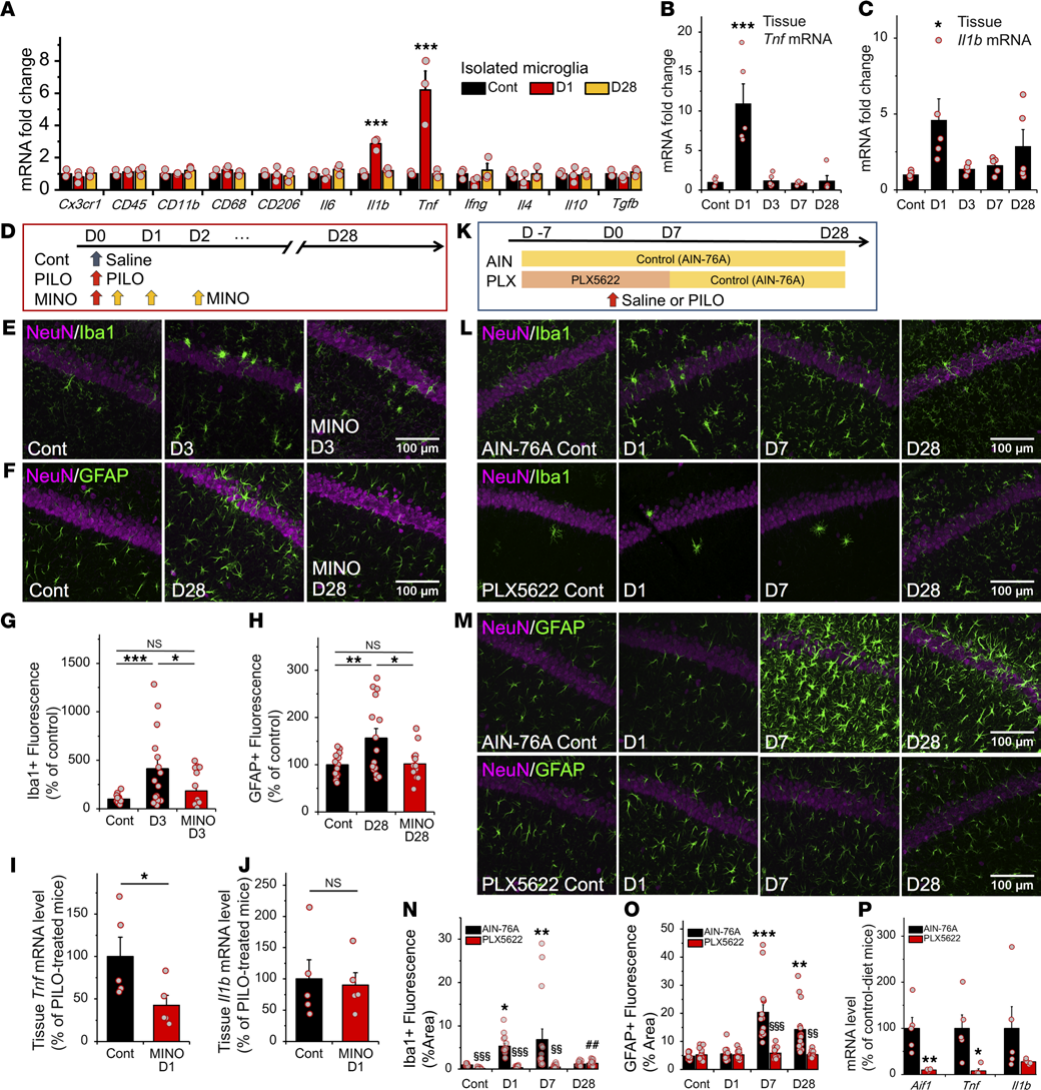
Microglia inhibition with minocycline and depletion with CSF1R antagonist (PLX5622) reduces astrogliosis. (**A**) Microfluidic quantitative RT-PCR analysis of mRNA in total RNA extracted from hippocampal microglia after SE (*n =* 3 samples/9 mice; ***P < 0.001 vs. control, 1-way ANOVA [*P* < 0.01, *P* < 0.001] with Dunnett’s test). (**B** and **C**) Quantitative RT-PCR analysis of mRNA in total hippocampal RNA after SE (*n =* 5 mice; *P < 0.05, ***P < 0.001 vs. control, 1-way ANOVA [*P* < 0.001, *P* < 0.05] with Dunnett’s test). (**D**) Experimental scheme for minocycline posttreatment-mediated microglia inhibition. (**E**–**H**) Representative microphotographs showing the spatiotemporal characteristics of Iba1 (**E**) and GFAP (**F**) expression and quantification of Iba1^+^ microglia (**G**) and GFAP^+^ astrocytes (**H**) in CA1 with or without minocycline after treatment after SE (*n =* 5 mice; NS *P* > 0.05, *P < 0.05, ***P < 0.001, 1-way ANOVA [*P* < 0.01] with Bonferroni test). (**I** and **J**) Quantitative RT-PCR analysis as in (**B** and **C**) with or without minocycline after treatment (*n =* 5 mice; NS *P* > 0.05, *P < 0.05, unpaired *t* test). (**K**) Experimental scheme for PLX5622-mediated microglia depletion. (**L**–**O**) Representative microphotographs showing the spatiotemporal characteristics of Iba1 (**L**) and GFAP (**M**) expression and quantification of Iba1^+^ microglia (**N**) and GFAP^+^ astrocytes (**O**) in CA1 with or without PLX5622 after SE (*n =* 5 mice; *P < 0.05, **P < 0.01 vs. control of AIN-76A [control diet]; ^##^*P* < 0.01 vs. control of PLX5622; ^§§^*P* < 0.01, ^§§§^*P* < 0.001 vs. AIN-76A [corresponding day]; 1-way ANOVA [*P* < 0.01] with Dunnett’s test and unpaired *t* test). (**P**) Quantitative RT-PCR analysis as in **B** and **C** with or without PLX5622 (*n =* 5 mice; *P < 0.05, **P < 0.01, unpaired *t* test).

Our data indicated temporal differences between activation of microglia and astrocytes, i.e., earlier and later onset after SE, respectively. To reveal features of the activated microglia after SE, we investigated the changes in mRNA levels of proinflammatory cytokines that are relevant to microglial activation by quantitative reverse transcription PCR (RT-PCR; Figure 3, A–C). SE increased *Tnf* and *Il1b* mRNA in the hippocampus on day 1 after SE (Figure 3, B and C). To explore the microglia-triggered astrocyte activation, we investigated microglial functional changes after SE. Among several molecules tested, we found that *Tnf* and *Il1b* mRNAs were also significantly upregulated in the isolated hippocampal microglia on day 1 after SE (Figure 3A).

To clarify whether microglial activation is required for astrogliosis, we investigated the effect of posttreatment with the inhibitor, minocycline (Figure 3D; refs. 37–39). To confirm the efficacy of minocycline in this protocol, microglial activation was assessed by IHC and quantitative RT-PCR. Minocycline posttreatment prevented the increase in the area of Iba1^+^ cells in CA1 on day 3 after the first SE (Figure 3, E and G) along with an increase in *Tnf* but not *Il1b* mRNA in the hippocampus on day 1 after the first SE (Figure 3, I and J). Notably, microglia inhibition with minocycline posttreatment prevented the increase in the area of GFAP^+^ cells in CA1 on day 28 after the first SE (Figure 3, F and H).

**Figure 4:**
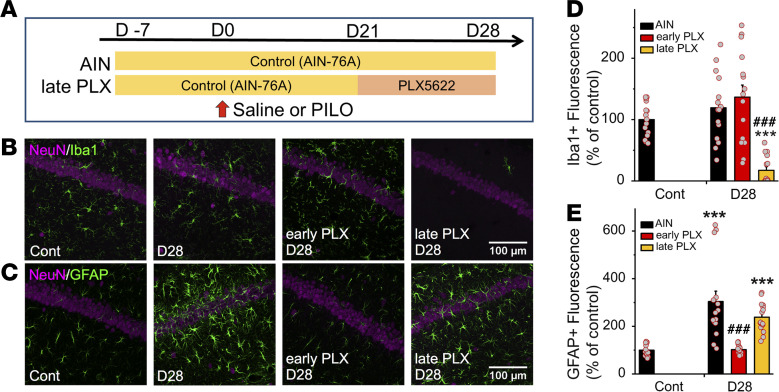
Microglia depletion with CSF1R antagonist (PLX5622) at late phase after SE does not reduce astrogliosis. (**A**) Experimental scheme for microglia depletion with PLX5622 at the late phase after SE. (**B** and **C**) Representative microphotographs showing the spatiotemporal feature of Iba1 (**B**) and GFAP (**C**) expression in CA1 with or without PLX5622 after SE. Fifteen images were collected per z-stack image (0.5 μm step). (**D** and **E**) Quantification of the temporal profile of Iba1^+^ microglia (**D**) and GFAP^+^ astrocytes (**E**) after SE (*n =* 5 mice; ***P < 0.01 vs. control, unpaired *t* test; ^###^*P* < 0.01 vs. AIN-76A [corresponding day], 1-way ANOVA [*P* < 0.001] with Dunnett’s test). Values represent mean ± SEM. Cont, control.

To further confirm that acute microglial activation plays an important role in the morphological activation of astrocytes after SE, we applied PLX5622, a CSF1R antagonist, to deplete microglia (Figure 3K; refs. 40–42). PLX5622 treatment prevented the increase in the area of Iba1^+^ cells in CA1 on days 1–7 after the first SE (Figure 3, L and N). In addition, *Aif1* and *Tnf* mRNA levels in PLX5622-treated mice were significantly decreased on day 1 after SE compared with those in the control diet mice (Figure 3P). Similarly, the increased area of GFAP^+^ astrocytes in CA1 from 7 to 28 days after SE in the control diet (AIN-76A) mice was prevented in PLX5622-treated mice (Figure 3, M and O). To identify the optimal timing of microglial inhibition to prevent astrogliosis, we applied PLX5622 3 weeks after SE (Figure 4A). This later PLX5622 treatment decreased the area of Iba1^+^ cells in CA1 on day 28 after the first SE (Figure 4, B and D) but did not prevent the increased area of GFAP^+^ astrocytes (Figure 4, C and E). These findings show that the initial reactive microglia were required to induce morphological activation of astrocytes after SE.

### Microglia inhibition reduced astrocytic Ca^2+^ hyperactivity.

**Figure 5:**
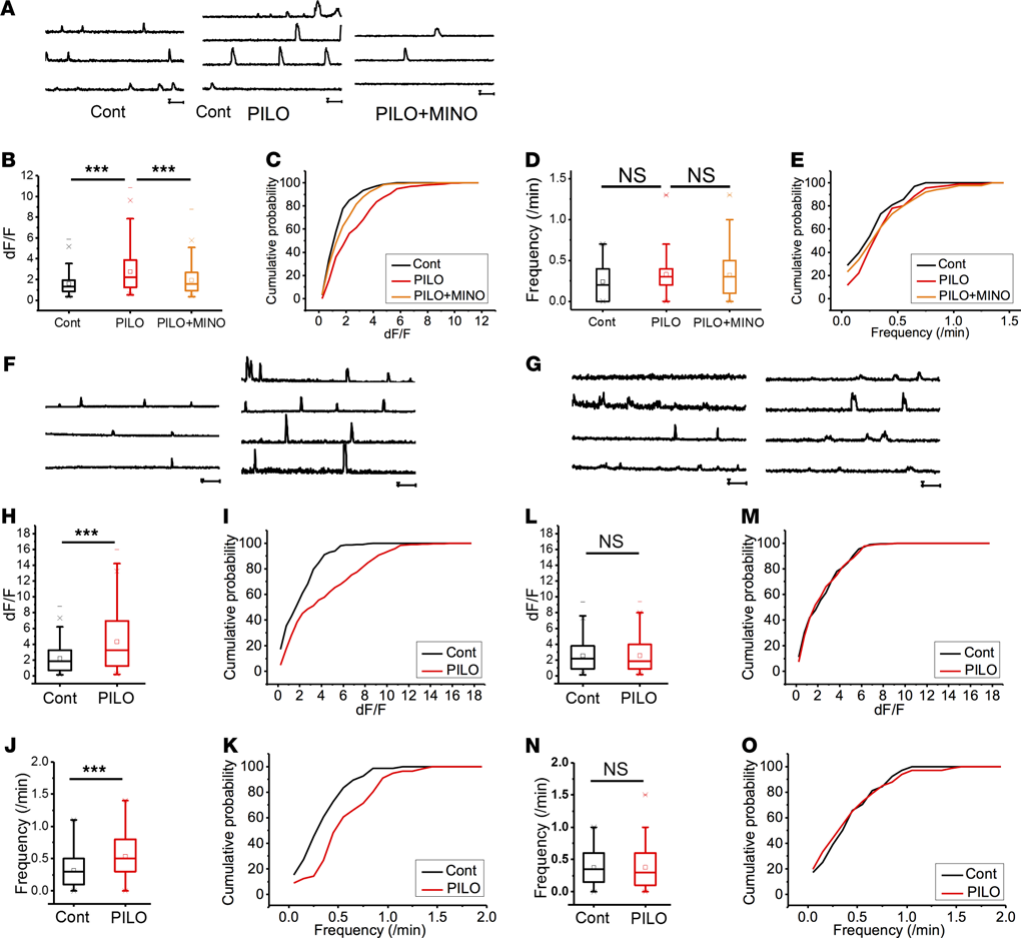
Microglia inhibition with minocycline or CSF1R antagonist (PLX5622) reduces the increased astrocytic Ca^2+^ hyperactivity after SE. (**A**) Ca^2+^ dynamics of astrocytes approximately 4 weeks after SE in the CA1 stratum radiatum region in Glast-CreERT2 flx-GCaMP3 mice with or without minocycline treatment. (**B**–**E**) Box plots showing Ca^2+^ signal amplitude (dF/F) (**B**) and frequency (**D**) (*n* = 3 mice and 74 cells [control], 92 cells [PILO], and 93 cells [PILO+MINO]; NS *P* > 0.05, ***P < 0.001, unpaired *t* test). Cumulative probability plots showing Ca^2+^ signal amplitude (dF/F) (*P* < 0.001, Kolmogorov–Smirnov test) (**C**) and frequency (NS *P* > 0.05, Kolmogorov–Smirnov test) (**E**). (**F** and **G**) Ca^2+^ dynamics of astrocytes approximately 4 weeks after SE in the CA1 stratum radiatum region in Glast-CreERT2 flx-GCaMP3 mice with (**G**) or without (**F**) PLX5622 treatment. (**H**–**K**) Box plots showing Ca^2+^ signal amplitude (dF/F) (**H**) and frequency (**J**) in the AIN-76A (control diet) group (*n* = 2 mice and 70 cells [control] and 58 cells [PILO]; ***P < 0.001, unpaired *t* test). Cumulative probability plots showing Ca^2+^ signal amplitude (dF/F) (*P* < 0.001, Kolmogorov–Smirnov test) (**I**) and frequency (*P* < 0.001, Kolmogorov–Smirnov test) (**K**) in the AIN-76A (control diet) group. (**L**–**O**) Box plots showing Ca^2+^ signal amplitude (dF/F) (**L**) and frequency (**M**) in the PLX5622 group (*n* = 2 mice and 61 cells [control] and 71 cells [PILO]; NS *P* > 0.05, unpaired *t* test). Cumulative probability plots showing Ca^2+^ signal amplitude (dF/F) (NS *P* > 0.05, Kolmogorov–Smirnov test) (**M**) and frequency (NS *P* > 0.05, Kolmogorov–Smirnov test) (**O**) in the PLX5622 group.

We then investigated whether microglial activation is required for astrocytic Ca^2+^ hyperactivity after SE. We also used a pharmacological approach to inhibit the early microglial activation after SE. Microglia inhibition with minocycline reduced the larger and frequent Ca^2+^ signals of astrocytes (Supplemental Video 5 and Figure 5, A–E). Similarly, the amplitude and frequency of Fluo-4AM–labeled astrocytic Ca^2+^ signaling after SE were significantly increased in the control diet (AIN-76A) mice (Figure 5, F, H, I, J, and K, and Supplemental Video 6). Conversely, the larger and frequent Ca^2+^ signals after SE were significantly reduced by the PLX5622 treatment (Figure 5, G, L, M, N, and O, and Supplemental Video 7). These results indicate that acute microglial activation was essential for the changes of astrocytic Ca^2+^ activity after SE.

### Microglia inhibition rescued enhanced seizure susceptibility.

**Figure 6:**
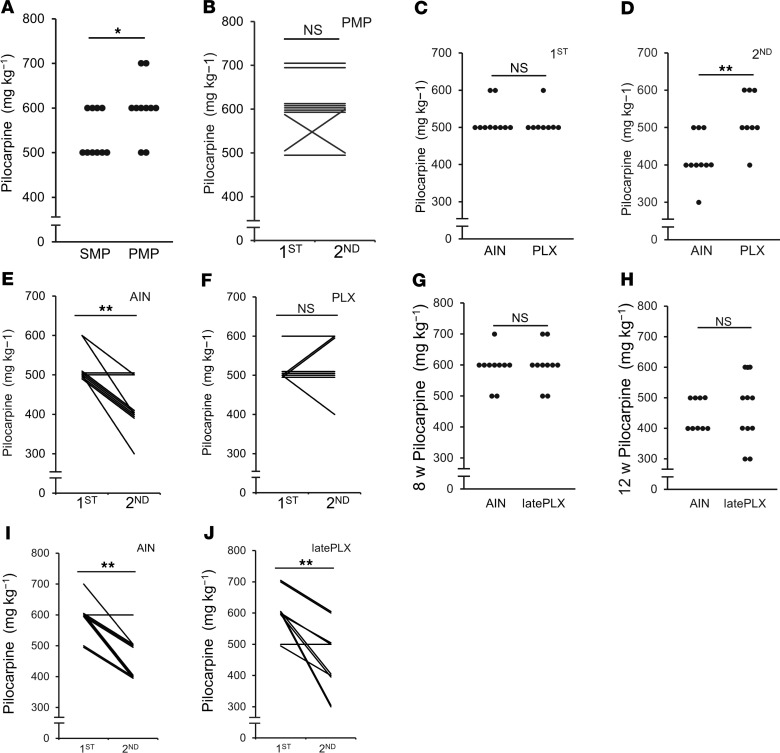
Microglia inhibition with minocycline or CSF1R antagonist (PLX5622) reduces the increased seizure susceptibility after SE. (**A**) Dot plots showing dose of pilocarpine required for the induction of the second SE (*n =* 10 mice; NS *P* > 0.05, *P < 0.05, Tied-collected Mann–Whitney *U* test). Mice were injected with saline with minocycline (SMP) at 8 weeks of age after treatment followed by an injection of pilocarpine with minocycline (PMP) at 12 weeks of age. (**B**) Scatter plot showing dose of pilocarpine required for the induction of the first (at 8 weeks of age) and second (at 12 weeks of age) SE (*n =* 10 mice; **P < 0.01, Wilcoxon signed-rank test). (**C** and **D**) Dot plots showing dose of pilocarpine required for the induction of the first (**C**) and second (**D**) SE with or without PLX5622 (*n* = 10 [AIN] and 8 [PLX] mice; NS *P* > 0.05, **P < 0.01, Mann–Whitney *U* test). AIN, control diet (AIN-76A). (**E** and **F**) Scatter plot showing dose of pilocarpine required for the induction of the first (at 8 weeks of age) and second (at 12 weeks of age) SE for AIN-76A (control diet) (**E**) or PLX5622 (**F**) (*n* = 10 [**E**] and 8 [**F**]; NS *P* > 0.05, **P < 0.01, Wilcoxon signed-rank test). (**G** and **H**) Dot plots showing dose of pilocarpine required for the induction of the first (**G**) and second (**H**) SE with or without late PLX5622 treatment (*n =* 10 mice; NS *P* > 0.05, Mann–Whitney *U* test). (**I** and **J**) Scatter plot showing dose of pilocarpine required for the induction of the first (at 8 weeks of age) and second (at 12 weeks of age) SE AIN-76A (control diet) (**I**) or PLX5622 (**J**) (*n =* 10 mice; **P < 0.01, Wilcoxon signed-rank test).

Finally, we tested whether microglia inhibition rescued the increased seizure susceptibility after SE. Of the 18 animals treated with pilocarpine and minocycline, 10 mice survived the treatment and received a second treatment (the lethality in the first SE was 44.4%). Posttreatment with minocycline after the first SE prevented the increased seizure susceptibility (Figure 6, A and B). No difference was observed between the control diet mice and the PLX5622-treated mice in the dose of pilocarpine required for the induction of the first SE (Figure 6C). Furthermore, there was no significant change in the number of interictal spikes in PLX5622-treated mice when compared with WT mice (Supplemental Figure 1F). These results indicate that microglia inhibition did not alter the acute responses to pilocarpine. In contrast, a lower dose of pilocarpine was required for the induction of the second SE in control mice compared with that in PLX5622-treated mice (Figure 6D). Consistent with this, unlike the enhanced seizure susceptibility observed in control mice after the first SE (as indicated by the reduced dose of pilocarpine required to induce the second vs. the first SE), there was no significant change in the dose of pilocarpine required for the induction of the first or second SE in PLX5622-treated mice (Figure 6, E and F). Of the 15 animals treated with pilocarpine and control diet, 10 survived and received a second treatment (the lethality in the first SE was 33.3%.). Of the 20 animals treated with pilocarpine and PLX5622, 10 survived and received a second treatment (the lethality in the first SE was 60.0%). In addition, interictal spikes were significantly reduced on days 7 and 28 after SE in PLX5622-treated mice compared with WT mice (Supplemental Figure 1, D and F). In contrast, a lower dose of pilocarpine was required for the induction of the second SE in mice with the later PLX5622 treatment, similar to that in the control diet mice (Figure 6, G–J). Of the 24 animals treated with pilocarpine and control diet, 10 survived and received a second treatment (the lethality in the first SE was 58.3%). Of the 17 animals treated with pilocarpine and PLX5622 later phase, 10 survived and received a second treatment (the lethality in the first SE was 41.2%). These data suggest that the inhibition of initial microglial activation rescued the increased seizure susceptibility.

## Discussion

Here, we demonstrate that SE induced sequential activation of glial cells, i.e., the initial activation of microglia, followed by astrocytic activation, which is essential for seizure susceptibility or epileptogenesis. The main findings in the present study are as follows: (a) microglia were activated and proinflammatory cytokines of microglia were increased immediately after SE; (b) reactive astrocytes, which exhibit larger IP_3_R2-mediated Ca^2+^ signals, appeared after microglial activation after SE; (c) genetic deletion of IP_3_R2 rescued both the aberrant Ca^2+^ signals in astrocytes and the increased seizure susceptibility; and (d) pharmacological inhibition of microglial activation or deletion of microglia at early phase after SE reduced astrogliosis along with aberrant Ca^2+^ signals of astrocytes and rescued the increased seizure susceptibility. These findings indicate that initially activated microglia were responsible for the subsequent induction of epileptogenic reactive astrocytes in vivo. The limitation of this study is that the severity of epilepsy was not evaluated by spontaneous recurrent seizures but was evaluated by changes in the threshold of pilocarpine-induced seizures and interictal spikes. However, overall our findings suggest that the therapeutic target to prevent epilepsy after SE should be shifted from microglia (early phase) to astrocytes (late phase).

Microglial and astrocytic activation is a common feature of various CNS disorders, including epilepsy (43–46). However, the pathological significance and spatiotemporal pattern of microglial and astrocytic activation in the epileptogenic process have not been carefully addressed. Microglial response to SE occurs immediately, with reactive microglia playing both detrimental and beneficial roles during acute seizures (47). Although activated microglia exhibit a neuroprotective role via the P2Y12 receptor in the acute phase, they exert proconvulsive effects through the production of proinflammatory cytokines such as IL-1β (11), TNF (48), and IL-6 (49, 50). However, such increase of purinergic receptors and proinflammatory cytokines after SE may be transient (11), and it is unknown how this transient microglial activation including proinflammatory cytokines causes long-term epileptic potential. Here, we found that inhibiting microglia at the acute phase (0–7 days after SE) but not the late phase (21–28 days after SE) reduced susceptibility to the second SE, suggesting that activated microglia triggered the epileptogenic process, including astrocytic activation, but did not exert a direct proconvulsive effect on the later phase after SE.

In the present study, we demonstrate that astrocytic activation developed slowly, starting on day 3 after SE, was long lasting, and was still observed when mice showed increased seizure susceptibility. Astrogliosis is thought to contribute to the pathophysiology of epilepsy (51–53). Some previous reports show dysregulation of astrocyte functions, such as K^+^ ion homeostasis (14), neurotransmitter buffering (15), gliotransmission (16), or purinergic signaling (54, 55), can actively contribute to hyperexcitation of neuronal networks and progression of seizures. However, the role of astrogliosis in epileptogenesis is largely unknown. In particular, it is important to determine whether activated astrocytes play a proconvulsive or anticonvulsive role in the epileptic brain. It has been proposed that astrocytic Ca^2+^ signaling contributes to the induction of epileptic seizures and neuronal cell loss by seizures (24, 31, 32, 56). In this study, we observed larger Ca^2+^ signals in the somatic regions of astrocytes in the latent phase of epileptogenesis. Analysis of the Ca^2+^ signals in astrocytes suggests that these Ca^2+^ signals are mediated by IP_3_R2. Notably, we found that genetic deletion of IP_3_R2 is sufficient to rescue the increased seizure susceptibility and reduce astrogliosis. Our study thus suggests that IP_3_R2-mediated Ca^2+^ signaling in reactive astrocytes played a proconvulsive role in the epileptic brain and could contribute to epileptogenesis.

Astrocytic Ca^2+^ signals may contribute to epileptogenesis through several mechanisms. Astrocytes impact neural circuit excitability directly by releasing “gliotransmitters,” such as glutamate (25, 57, 58). Astrocytes also increase neuronal excitability by forming new circuits through the release of synaptogenic molecules (23, 59). However, the functional consequences of these changes in the context of epileptogenesis remain to be determined. As Ca^2+^ ions serve as a ubiquitous intracellular signal in the regulation of numerous cellular processes, including exocytosis, proliferation, and gene expression, it is also likely to regulate many processes in the induction or maintenance of reactive astrocytes (60, 61). Since it has been reported that the Ca^2+^ signals in astrocytes can contribute to ictogenesis (27, 29), we cannot disregard the possibility that IP_3_R2 may contribute to neural excitability and microglial activation after SE. We demonstrate that SE induced neither an increase in Ca^2+^ excitation in astrocytes nor proconvulsive effects in IP_3_R2-KO mice, suggesting that enhanced Ca^2+^ signals in astrocytes were likely responsible for epileptogenesis.

In animal models of epilepsy, reactive astrocytes undergo extensive physiological changes involving not only Ca^2+^ signaling but also ion and neurotransmitter homeostasis along with intracellular and extracellular water content, which can cause neuronal hyperexcitability (17, 62–64). The relative importance of such functional changes of astrocytes to epileptogenesis will be investigated in future studies. Recently, it has been reported that activated microglia can induce neurotoxic reactive astrocytes (i.e., A1 astrocytes), which release unidentified neurotoxic factors (41, 65). Thus, whether astrogliosis after SE results in a similar phenotype to A1 astrocytes and whether IP_3_-mediated Ca^2+^ signals contribute to the induction of neurotoxic phenotype (61) represent relevant issues to be addressed in future investigations. However, it was also reported that these functional changes of astrocytes, including gap junction dysfunction (17), could occur before the increase is observed in GFAP immunostaining, astrocytic Ca^2+^ signals, or Iba1 immunostaining investigated in this study. Although whether the astrocytes induced by activated microglia are in a primarily neurotoxic or neuroprotective state remains largely unknown, our data suggest that the reactive astrocytes induced by activated microglia after SE exerted proconvulsive effects in the epileptic brain.

In this study, we also demonstrate that proinflammatory cytokines of microglia were increased prior to astrocytic activation, suggesting the importance of microglial activation as an initial process of epileptogenesis. Pharmacological inhibition and depletion of microglia significantly blocked the activation of astrocytes and decreased the seizure threshold after SE. Our findings identify that activated microglia likely promoted epileptogenesis by inducing the proconvulsive phenotype of astrocytes. Although it has been recognized that microglial activation occurs before reactive astrogliosis in various CNS diseases (66–68), little was known prior to the present study regarding how microglial–astrocytic interactions contribute to the pathophysiology of epilepsy. For example, several previous studies using chemoconvulsant-induced epilepsy models have shown that activated microglia were present immediately after SE and that functional changes occurred, such as upregulation of proinflammatory cytokines (8, 69, 70), purinergic receptors (43), and phagocytosis (44).

Previous reports also revealed that microglia modulate astrocyte activation via various molecules, especially proinflammatory cytokines (71, 72). Consistent with this, we found that TNF and IL-1β were significantly upregulated in hippocampal microglia on day 1 after SE. Conversely, microglia inhibition by minocycline prevented the increased mRNA of TNF in the hippocampus on day 1 after the first SE along with subsequent reactive astrogliosis, suggesting a potential role of proinflammatory cytokines from microglia in reactive astrogliosis after SE. Because the effect of minocycline may not be restricted to microglia, we depleted microglia using a CSF-1 receptor antagonist and found similar results, suggesting that microglial activation occurred through cytokine release. CSF1 receptor antagonist may affect not only microglia but also peripheral macrophages (73, 74), which can contribute to pathophysiology of epilepsy (75–77). Thus, despite the potential problem of specificity owing to the use of pharmacological inhibition of microglia, we clearly show that initial activation of microglia and microglia-derived proinflammatory cytokines likely underlay the subsequent astrogliosis-mediated epileptogenesis. Nevertheless, because the molecular mechanisms underlying the activation of astrocytes triggered by activated microglia have not been fully clarified, other chemical mediators such as ATP may also contribute to activate microglia-mediated astrogliosis (78). Further investigations using more specific interventions are required to elucidate the precise molecular mechanisms underlying the interaction between microglia and astrocytes.

In summary, our findings identify a sequence of glial activation in the hippocampus that contributed to the epileptogenic process. In this process, microglial activation was identified as a crucial event to induce reactive astrocytes. In turn, astrocytic Ca^2+^ activation, mediated by IP_3_R2, played an important role in the induction of epileptogenesis. Our findings add to the emerging view that reactive astrocytes triggered by microglia have a central role in the pathogenesis of epilepsy and, given the limited progress of neuron-centered epilepsy research over the past several years, suggest reactive glial cells as promising targets for the development of alternative and more specific antiepileptic drugs.

## Methods

### Animals.

All studies used male C57BL/6J mice (Japan SLC Inc.). IP_3_R2-KO mice on a C57BL/6 background were available from a previous study (36); their generation and maintenance have been previously described in detail. Glast-CreERT2 flx-GCaMP3 mice on a C57BL/6 background were also available from a previous study (34, 35); their generation and maintenance have been previously described in detail. In the present study, we performed IHC and confirmed that GCaMP3 was colocalized with GFAP, an astrocyte marker, but not with Iba1 or NeuN (Supplemental Figure 3 and Supplemental Table 1). Overall, Ca^2+^ signals detected by GCaMP3 were mainly detected from astrocytes.

Mice were housed on a 12-hour light (6 am)/dark (6 pm) cycle with ad libitum access to water and rodent chow. The animals were allowed to adapt to laboratory conditions for at least 1 week before starting the experiments.

### Animal treatments.

The first SE was induced in 8-week-old male mice by the administration of pilocarpine and the second SE was induced 4 weeks after the first SE. A low dose of 100 mg/kg pilocarpine (161-07201, Wako) per injection was administered i.p. every 20 minutes until the onset of Racine scale stage 5 seizures. Scopolamine methyl bromide (1 mg/kg, i.p.; 198-07971, Wako) was administered 30 minutes prior to pilocarpine injection to reduce its peripheral effects (32, 33). Seizures were terminated with pentobarbital (20 mg/kg, i.p.; Kyoritu Seiyaku) when mice experienced stage 5 seizures for 30 minutes. Behavior of pilocarpine-treated mice was observed for 1 hour after SE. To examine whether the first SE increased seizure susceptibility, the second SE was induced 4 weeks after the first SE using the same protocol.

To establish whether minocycline inhibits acute seizure-induced microglial activation, mice were administered i.p. with saline or minocycline (25 mg/kg) 1 hour after pilocarpine-SE induction and for the following 2 consecutive days (37–39). Microglia were also depleted from mice by treatment with the CSF1R antagonist, PLX5622 (Plexxikon), formulated in AIN-76A rodent chow (Research Diets). Mice were treated with PLX5622 (1200 mg/kg Chow) or a matched control diet (AIN-76A) for 7 days before SE and the following 7 consecutive days (40–42).

### EEG acquisition.

The mice were deeply anesthetized with isoflurane. For EEG recordings, a bipolar electrode was implanted at the left CA1 area of the dorsal hippocampus (AP = –1.8 mm, ML = +1.6 mm, DV = –2.0 mm). The electrode was fixed to the skull with dental cement. Animals were allowed to recover for 5–7 days before EEG recording. EEGs were recorded in freely moving mice using a digital acquisition system (PowerLab 26T, ADInstruments), for at least 2 hour per day. EEG data were collected at a sampling rate of 2000 Hz. Data were acquired, digitized, and analyzed offline using Labchart 8 software (ADInstruments). The artifacts in the raw EEG traces were manually identified and excluded from the analyses of interictal spikes.

### IHC.

The mice were deeply anesthetized with pentobarbital and perfused transcardially with PBS, followed by 4% (w/v) paraformaldehyde in PBS. The brains were removed, postfixed overnight, and then cryoprotected with 30% (w/v) sucrose in PBS for 2 days. The brains were frozen and coronal sections (20 μm) were cut using a cryostat (CM1100, Leica). Slices were washed with PBS 3 times and treated with 0.1% Triton X-100/10% NGS for 1 hour to block nonspecific binding. The sections were incubated for 2 days at 4°C with the following primary antibodies: monoclonal rat anti-GFAP (1:2000; 13-0300, ThermoFisher Scientific), monoclonal mouse anti-NeuN (1:500; Millipore, MAB377), polyclonal rabbit anti-Iba1 (1:1000; Wako, 019-19741), polyclonal chicken anti-GFP antibody (1:1000; A10262, ThermoFisher Scientific), and monoclonal rabbit anti-NeuN (1:1,000; MABN140, Millipore). The sections were washed 3 times with PBS, then incubated for 2 hours at room temperature with the following secondary antibodies: Alexa 488– or Alexa 546–conjugated polyclonal goat anti-mouse, -rat, -rabbit, or -chicken IgGs (1:500; A11029, A-11081, A11035, A11039, ThermoFisher Scientific). After washing slices 3 times with PBS, they were mounted with Vectashield Mounting Medium (Vector Laboratories). Fluorescence images were obtained using a confocal laser microscope system (FV-1000; Olympus) or Keyence fluorescence microscope (BZX-700).

### Standard quantitative RT-PCR.

Total RNA was isolated and purified from tissues using the RNeasy Lipid Tissue Mini Kit (QIAGEN) according to the manufacturer’s instructions. RT-PCR amplifications were performed using the One Step PrimeScript RT-PCR Kit (TaKaRa Bio). RT-PCR amplifications and real-time detection were performed using an Applied Biosystems 7500 Real-Time PCR System. The thermocycling parameters were as follows: 5 minutes at 42°C for reverse transcription, 10 seconds at 95°C for inactivation of the reverse transcriptional enzyme), and 40 cycles of denaturation (5 seconds at 95°C) and annealing or extension (34 seconds at 60°C). Relative gene expression was calculated using *Gapdh* expression as a housekeeping gene. All primer probe sets and reagents were purchased from Applied Biosystems: rodent *Gapdh* (4308313), mouse *Tnf* (Mm00443260_g1), mouse *Il1b* (Mm00434228_m1).

### Dissociated cell suspensions from adult mouse brain.

Three 8-week-old male mice were perfused with PBS after anesthesia to eliminate serum vesicles and hippocampi were dissected to comprise 1 sample. Tissue dissociation was performed using the gentleMACS dissociator and the Adult Brain Dissociation Kit (Miltenyi Biotec) according to the manufacturer’s protocol. Briefly, brain tissue was minced and digested with a proprietary enzyme solution on the gentleMACS dissociator adult brain program. The cells were then incubated with anti-mouse CD11b–coated microbeads (Miltenyi Biotec) for 10 minutes at 4°C. The cell-bead mix was then washed to remove unbound beads. Prior to antibody labeling, nonspecific binding to the Fc receptor was blocked using the FcR Blocking Reagent (Miltenyi Biotec). Cells were suspended in PBS with 0.5% BSA and the cell suspension was loaded onto an LS Column (Miltenyi Biotec), which was placed in the magnetic field of a QuadroMACS Separator (Miltenyi Biotec). The magnetically labeled CD11b^+^ cells were retained within the column and eluted as the positively selected cell fraction after removing the column from the magnet.

### Microfluidic quantitative RT-PCR.

Total RNA was extracted from dissociated cells using the RNeasy Lipid Tissue Mini Kit (QIAGEN) and cDNA synthesis performed using the PrimeScript RT-PCR Kit (Perfect Real Time; TaKaRa Bio). For preamplification, up to 100 qPCR assays (primer or probe sets in 20× stock concentration) were pooled and diluted to a 0.2× concentration. For microfluidic qPCR, 1.25 μL of each cDNA sample was preamplified using 1 μL of TaqMan PreAmp Master Mix (PN 100-5580, Fluidigm), 1.25 μL of the primer pool, and 1.5 μL of water. Preamplification was performed using a 2-minute 95°C denaturation step and 14 cycles of 15 seconds at 95°C and 4 minutes at 60°C. Microfluidic quantitative RT-PCR reactions were performed using the 96 × 96 chips and included 2–3 technical replicates for each combination of sample and assay. For sample mixtures, 2.7 μL preamplification product was combined with 0.3 μL of 20X GE Sample Loading Reagent (85000746, Fluidigm) and 3 μL of 2X PCR Master Mix (4324020, ThermoFisher Scientific), 5 μL of which was loaded into sample wells. For assay mixtures, equal volumes of TaqMan assay and 2X Assay Loading Reagent (PN85000736, Fluidigm) were combined, and 5 μL of the resulting mixture was loaded into multiple assay wells. RT-PCR amplifications and real-time detection were performed using the BioMarkHD Real-Time PCR System (Fluidigm). Data from Fluidigm runs were manually checked for reaction quality prior to analysis, and Ct values for each gene target were normalized to Ct values for housekeeping genes. All primer probe sets and reagents were purchased from Integrated DNA Technologies: rodent *Gapdh* (Mm.PT.39a.1), mouse *Tnf* (Mm.PT.58.12575861), mouse *Il1b* (Mm.PT.58.41616450), mouse *Cx3cr1* (Mm.PT.58.17555544), mouse *CD45* (Mm.PT.58.7583849), mouse *CD11b* (Mm.PT.58.14195622), mouse *CD68* (Mm.PT.58.32698807), mouse *CD206* (Mm.PT.58.42560062), mouse *Il6* (Mm.PT.58.10005566), mouse *Ifng* (Mm.PT.58.41769240), mouse *Il4* (Mm.PT.58.32703659), mouse *Il10* (Mm.PT.58.13531087), and mouse *Tgfb* (Mm.PT.58.11254750).

### Preparation of brain slices and Ca^2+^ imaging.

The methods used have been described previously (61, 79). Briefly, 8-week-old male mice were anesthetized with pentobarbital (100 mg/kg, i.p.). Cold cutting ACSF, composed of 92 mM NaCl, 2.5 mM KCl, 1.2 mM NaH_2_PO_4_, 30 mM NaHCO_3_, 20 mM HEPES, 25 mM D-glucose, 5 mM sodium ascorbate, 2 mM thiourea, 3 mM sodium pyruvate, 10 mM MgCl_2_, and 0.5 mM CaCl_2_ saturated with 95% O_2_ and 5% CO_2_, was perfused transcardially. Coronal slices of the hippocampus (300 μm) were cut using a vibrating microtome (Pro7, Dosaka) in cutting ACSF. Slices were incubated at 34°C for 10 minutes in recovery ACSF, composed of 93 mM N-methyl-D-glucamine, 93 mM HCl, 2.5 mM KCl, 1.2 mM NaH_2_PO_4_, 30 mM NaHCO_3_, 20 mM HEPES, 25 mM D-glucose, 5 mM sodium ascorbate, 2 mM thiourea, 3 mM sodium pyruvate, 10 mM MgCl_2_, and 0.5 mM CaCl_2_ saturated with 95% O_2_ and 5% CO_2_, and subsequently stored in ACSF comprising 124 mM NaCl, 2.5 mM KCl, 1.2 mM NaH_2_PO_4_, 24 mM NaHCO_3_, 5 mM HEPES, 12.5 mM D-glucose, 5 mM sodium ascorbate, 2 mM thiourea, 3 mM sodium pyruvate, 2 mM MgCl_2_, and 2 mM CaCl_2_ saturated with 95% O_2_ and 5% CO_2_ at room temperature. After 1 hour of recovery, slices were submerged in ACSF at approximately 32°C. Slices were imaged using an Olympus Fluoview FV1000MPE 2-photon laser scanning microscope equipped with a Maitai HP DS-OL laser (Spectra-Physics). We used a 920 nm laser and 495–540 nm bandpass emission filter. Astrocytes were selected from the CA1 stratum radiatum region and were typically 30–50 μm from the slice surface. Images were gathered using a 40× water immersion lens (Olympus) with a numerical aperture of 0.80.

For Fluo-4AM measurements, we dropped 2.5 μL Fluo-4AM (2 mM) onto the hippocampal slices followed by incubation in ACSF for 60 minutes, then transferred the slices to dye-free ACSF for at least 30 minutes prior to experimentation. The final concentration of Fluo4-AM was 5 μM with 0.02% Pluronic F–127. Astrocytes were selected from the CA1 stratum radiatum region and were typically 30–50 μm from the slice surface. TTX (1 μM), 2-APB (100 μM), and CPA (20 μM) were solubilized in ACSF. Baseline astrocytic activity was recorded prior to drug application. Subsequently, drugs were applied onto the slice for 10 minutes and astrocytic activity was recorded for 10 minutes.

### Image analysis.

Images were acquired using inverted confocal laser scanning systems (Olympus FV-1000) (original magnification, ×40) with a 1.30 numerical aperture objective lens. Information regarding z-stack images is described in the figure legends. Astrocytes were selected from the CA1 stratum radiatum region and imaged based on GFAP immunostaining. Microglia were imaged based on Iba1 immunostaining at the CA1 stratum radiatum region. Subsequent images were processed and quantified using NIH ImageJ software. For the quantitative analysis of the area containing Iba1^+^ microglia, we randomly chose 3 fields per mouse. Images were converted to gray scale and the quantification threshold was set constantly for all specimens within each experimental group. The percentage of Iba1^+^ area was calculated by dividing the area of Iba1^+^ region by the total area of the region of interest. For the quantitative analysis of the area containing GFAP^+^ astrocyte, the percentage of GFAP^+^ area was calculated using the same method used to quantify Iba1^+^ microglia.

The methods used for Ca^2+^ imaging data analysis have been described previously (56, 70). Briefly, imaging data were analyzed using NIH ImageJ software. We selected regions of interest from somatic regions of astrocytes by visual examination of the time lapse image. Using these regions of interest, raw fluorescence intensity values (F) were taken from the original videos and converted to delta F/F (dF/F) in Originlab (Origin Lab Corp.). We analyzed Ca^2+^ signals when their dF/F values were greater than 0.2. We analyzed Ca^2+^ signals and their amplitude (dF/F) and duration (full width at half maximum) using the Originlab “peak analysis” function.

### Statistics.

All statistical analyses were performed using SPSS version 19.0 (SPSS Inc.) software. Data are presented as mean ± SEM. Most data were analyzed using 1-way ANOVA followed by Dunnett’s multiple post hoc test for comparing more than 3 samples, and 2-sample unpaired *t* tests. All *t* tests were 2-tailed tests. A *P* value of less than 0.05 was considered statistically significant.

### Study approval.

All experimental procedures were performed in accordance with ref. 80 and with the previous approval of the Animal Care Committee of the University of Yamanashi.

## Author contributions

FS and SK conceived and designed the research. FS performed most of the experiments, analyzed the data, and wrote the manuscript. HT contributed to the IHC experiments. K. Saito contributed to the MACS experiments. YS, ES, and SK analyzed the data. KM provided IP3R2-KO mice. HH, DLC, and JN contributed to the EEG experiments. K. Sugita, MA, and SK supervised the project. All of the authors discussed and commented on the manuscript.

## Supplementary Material

Supplemental data

Supplemental Video 1

Supplemental Video 2

Supplemental Video 3

Supplemental Video 4

Supplemental Video 5

Supplemental Video 6

Supplemental Video 7

## Version 1 04/08/2021

In-Press Preview

## Version 2 05/10/2021

Electronic publication
