# Supplementary material for: Reactive astrocyte-driven epileptogenesis is induced by microglia initially activated following status epilepticus
Source: JCI Insight. 2021 May 10;6(9):e135391. doi: 10.1172/jci.insight.135391 (PMC8262323; doi:10.1172/jci.insight.135391)
Supplement: Supplemental data [file jciinsight-6-135391-s252.pdf]

Supporting information

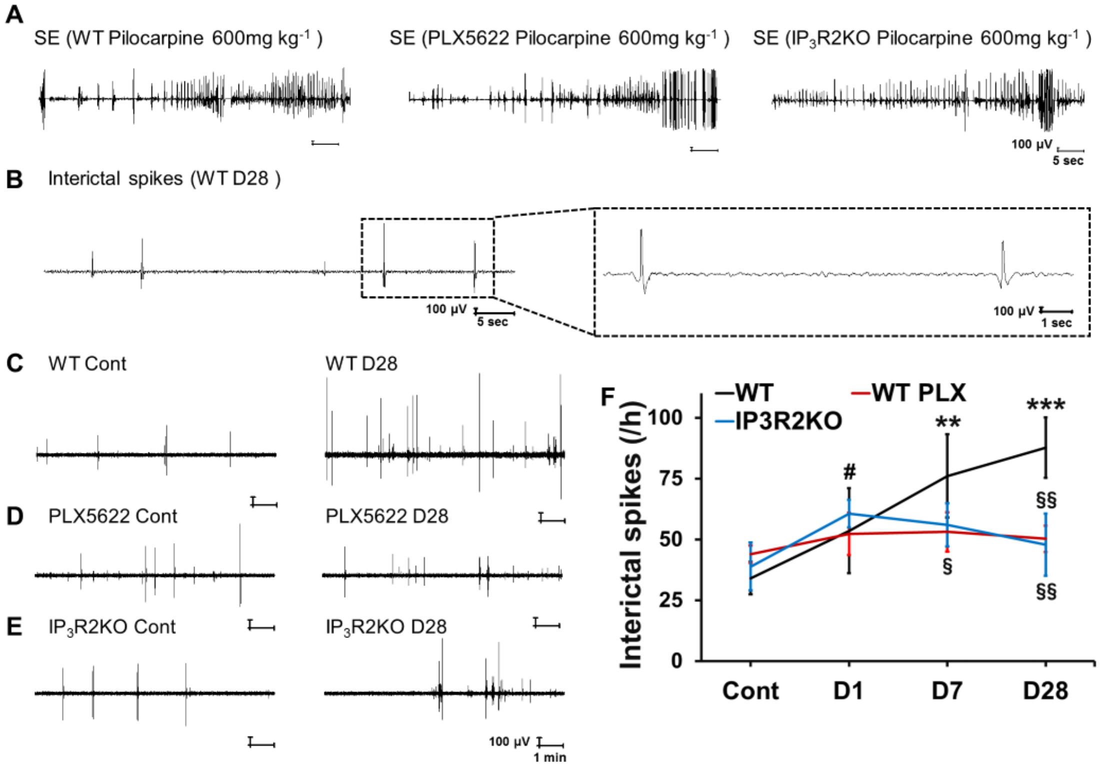

S1 Fig Microglia depletion with CSF1R antagonist (PLX5622) or IP_3R2KO mice reduces the increased interictal spikes following SE.

(A) Sample EEG from a WT, PLX5622-treated and IP_3R2 knockout mouse during a pilocarpine-induced stage 5 seizure. (B) Sample EEG presenting Interictal spikes from a WT mouse at 28 days after SE. (C) Interictal spikes (for 10 min) in WT control and 28 days after SE. (D) Interictal spikes (for 10 min) in WT control and 28 days after SE with PLX5622 treatment. (E) Interictal spikes (for 10 min) in IP_3R2KO mice control and 28 days after SE. (F) Quantification of the temporal profile of interictal spikes after SE (n = 5 mice, $^{**}P < 0.01$, $^{***}P <$

0.001 vs. control of WT mice, $^{\#}P < 0.05$ vs. control of PLX5622, $^{\S}P < 0.05$, $^{\S\S}P < 0.01$ vs. WT (corresponding day), one-way ANOVA ($P < 0.01$) with Dunnett's test and unpaired t-test). Values represent the means ± SEM. Cont, control; D, day.

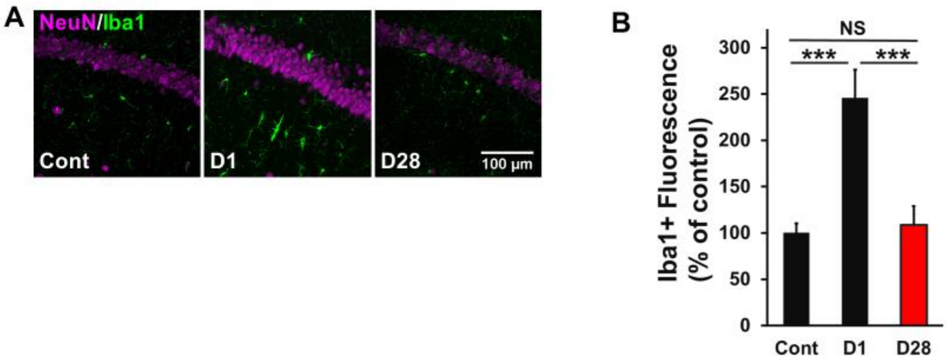

S2 Fig Initial microglial activation is observed after SE in IP3R2KO mice.

(A) Representative microphotographs showing the spatiotemporal characteristics of Iba-1 expression in CA1 after SE. Fifteen images were captured per z-stack image (0.5 μm step). (B) Quantification of the temporal profile of Iba-1 positive microglia in IP3R2KO mice after SE (n = 4 mice, N.S. means not significant ($P > 0.05$), ***$P < 0.001$, one-way ANOVA ($P < 0.001$) with Bonferroni test). Values represent the mean ± SEM. Cont, control; D, day.

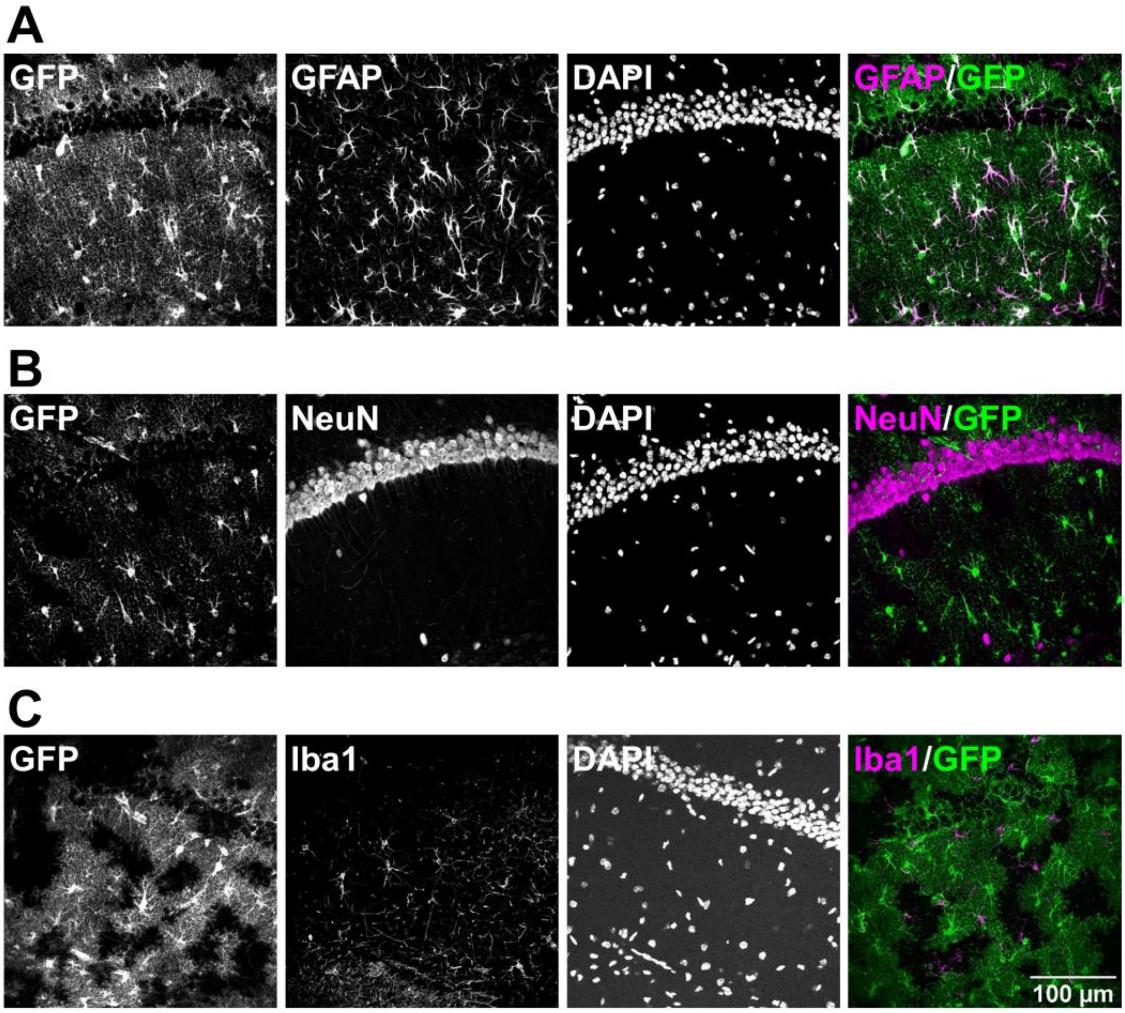

S3 Fig Immunohistochemical analysis of GCaMP expression in the hippocampus in Glast-CreERT2::Flx-GCaMP3 mice.

(A to C) Representative images showing immunohistochemical staining for GFAP (A), NeuN (B), and Iba1 (C) with GFP staining in the CA1 region of Glast-CreERT2::Flx-GCaMP3 mice (tamoxifen i.p. at P7).

S1 Table Cell-specific markers in GCaMP3-expressing cells in the hippocampus of Glast-CreERT2::Flx-GCaMP3 mice (tamoxifen i.p. at P7).

Region	GFAP/GFP	NeuN/GFP	Iba1/GFP
CA1	92 ± 2%<br>(12 FOV/6 slices)	0 ± 0%<br>(12 FOV/6 slices)	0 ± 0%<br>(12 FOV/6 slices)
CA2	86 ± 5%<br>(6 FOV/6 slices)	0 ± 0%<br>(6 FOV/6 slices)	0 ± 0%<br>(6 FOV/6 slices)

n = 3 mice, values represent the means ± SEM. FOV, fields of view.
